# Prevention of Unplanned Hospital Admissions in Multimorbid Patients Using Computational Modeling: Observational Retrospective Cohort Study

**DOI:** 10.2196/40846

**Published:** 2023-02-16

**Authors:** Rubèn González-Colom, Carmen Herranz, Emili Vela, David Monterde, Joan Carles Contel, Antoni Sisó-Almirall, Jordi Piera-Jiménez, Josep Roca, Isaac Cano

**Affiliations:** 1 Hospital Clínic de Barcelona Institut d’Investigacions Biomèdiques August Pi i Sunyer (IDIBAPS) Universitat de Barcelona Barcelona Spain; 2 Consorci d'Atenció Primària de Salut Barcelona Esquerra (CAPSBE) Primary Healthcare Transversal Research Group Institut d'Investigacions Biomèdiques August Pi i Sunyer (IDIBAPS) Barcelona Spain; 3 Catalan Health Service Barcelona Spain; 4 Digitalization for the Sustainability of the Healthcare System DS3-IDIBELL L’Hospitalet de Llobregat Spain; 5 Catalan Institute of Health Barcelona Spain; 6 Health Department Generalitat de Catalunya Barcelona Spain; 7 Faculty of Informatics, Multimedia and Telecommunications Universitat Oberta de Catalunya Barcelona Spain

**Keywords:** health risk assessment, health risk profiles, transitional care, hospital readmissions, mortality

## Abstract

**Background:**

Enhanced management of multimorbidity constitutes a major clinical challenge. Multimorbidity shows well-established causal relationships with the high use of health care resources and, specifically, with unplanned hospital admissions. Enhanced patient stratification is vital for achieving effectiveness through personalized postdischarge service selection.

**Objective:**

The study has a 2-fold aim: (1) generation and assessment of predictive models of mortality and readmission at 90 days after discharge; and (2) characterization of patients’ profiles for personalized service selection purposes.

**Methods:**

Gradient boosting techniques were used to generate predictive models based on multisource data (registries, clinical/functional and social support) from 761 nonsurgical patients admitted in a tertiary hospital over 12 months (October 2017 to November 2018). K-means clustering was used to characterize patient profiles.

**Results:**

Performance (area under the receiver operating characteristic curve, sensitivity, and specificity) of the predictive models was 0.82, 0.78, and 0.70 and 0.72, 0.70, and 0.63 for mortality and readmissions, respectively. A total of 4 patients’ profiles were identified. In brief, the reference patients (cluster 1; 281/761, 36.9%), 53.7% (151/281) men and mean age of 71 (SD 16) years, showed 3.6% (10/281) mortality and 15.7% (44/281) readmissions at 90 days following discharge. The unhealthy lifestyle habit profile (cluster 2; 179/761, 23.5%) predominantly comprised males (137/179, 76.5%) with similar age, mean 70 (SD 13) years, but showed slightly higher mortality (10/179, 5.6%) and markedly higher readmission rate (49/179, 27.4%). Patients in the frailty profile (cluster 3; 152/761, 19.9%) were older (mean 81 years, SD 13 years) and predominantly female (63/152, 41.4%, males). They showed medical complexity with a high level of social vulnerability and the highest mortality rate (23/152, 15.1%), but with a similar hospitalization rate (39/152, 25.7%) compared with cluster 2. Finally, the medical complexity profile (cluster 4; 149/761, 19.6%), mean age 83 (SD 9) years, 55.7% (83/149) males, showed the highest clinical complexity resulting in 12.8% (19/149) mortality and the highest readmission rate (56/149, 37.6%).

**Conclusions:**

The results indicated the potential to predict mortality and morbidity-related adverse events leading to unplanned hospital readmissions. The resulting patient profiles fostered recommendations for personalized service selection with the capacity for value generation.

## Introduction

Enhanced management of multimorbidity constitutes a major clinical challenge because the number and complexity of morbid conditions show well-established causal relationships with mortality and high use of health care resources [1], with unplanned hospital admissions being a key determinant of the multimorbidity-induced high burden on health care systems worldwide [2,3].

Evidence-based efficacy of integrated care interventions to prevent hospitalizations in high-risk patients has been demonstrated [4,5]. Likewise, comprehensive programs to enhance care during transitions after hospital discharge can reduce all-cause early hospital readmissions in chronically ill patients, which is particularly effective in mid/long-term evaluations [6,7]. However, the scalability and adoption of such preventive interventions in real-life scenarios are often limited by an efficacy-effectiveness gap. Poor patient risk stratification and insufficient workforce preparation for the care continuum have been identified as critical limiting factors for effective clinical practice [8].

In this regard, multisource clinical predictive modeling approaches, considering various determinants of health (eg, clinical, social, populational, lifestyle), have become an effective strategy for subject-specific risk assessment to prevent morbidity-related adverse events leading to hospital readmissions [9-13].

This research work aimed to enhance chronic patients’ stratification at hospital discharge, characterize patients’ risk profiles for generating recommendations on postdischarge care transitions [14,15], and improve personalized preventive care pathways within a care continuum scenario [16]. To this end, multiple data sources (ie, primary care, social care, hospital-based data, and registry information) from distinct domains (ie, medical complexity, disability scoring, unhealthy lifestyle factors, and social frailty) have been considered.

## Methods

### Study Design, Population, Potential Predictors, and Data Sources

This is an observational retrospective cohort study of patients discharged from the Hospital Clínic of Barcelona (HCB) from October 2017 to November 2018. The study population included nonsurgical patients admitted to the hospital avoidance program (n=441) and the corresponding controls undergoing conventional hospitalization (n=441), as reported in detail in Herranz et al [17].

Key determinants of health from the clinical and social domains were considered (Table 1): (1) sociodemographic information; (2) population-based registry indicators on morbidity and complexity; (3) patients’ functional characteristics; (4) frailty and social risk indicators; (5) unhealthy lifestyle habits; (6) utilization of health care resources; (7) clinical and biological data collected during the acute episode; and (8) immunization records. It is of note that multimorbidity and complexity were characterized by the Catalan population–based health risk assessment scoring, known as Adjusted Morbidity Groups (AMG) [18-21], an aggregative index that indicates the burden of an individual’s morbid conditions through a disease-specific weighting deduced from statistical analysis based on mortality and the utilization of health services; by contrast, the acute episode complexity was characterized by the Queralt Indices [22,23] that combine information on (1) preexisting comorbidities; (2) in-hospital complications; (3) principal discharge diagnoses; (4) main procedure; and (5) secondary procedures performed during hospitalization. For predictive modeling purposes, the different Queralt Indices have been aggregated into a single score, referred to as the composite Queralt Index.

**Table 1:** List of variables considered for predictive modeling and clustering analysis.^a^

Variables		Description
**Sociodemographic data**		
	1: Age (100%)	Patient’s age (numerical)
	1: Gender (100%)	Patient’s sex (binary, male/female)
	1: HBA (100%)	Health Basic Area (categorical, 105 levels)
**Medical complexity**		
	3: AMG score [18-21] (100%)	Adjusted Morbidity Groups score (numerical)
	2: SIIP Plan [24] (90.45%)	Shared Individual Intervention Plan (binary, yes/no)
	2: CCP [24] (90.45%)	Complex chronic patient (binary, yes/no)
	2: ACP [24] (90.45%)	Advanced chronic patient (binary, yes/no)
**Patient’s functional capacity**		
	2: Barthel [25] (90.45%)	Barthel Index (numerical, 0-100)
	2: Lawton Brody [26] (82.97%)	Lawton Brody Index (numerical, 0-8)
	2: Pfeiffer [27] (90.58%)	Pfeiffer Index (numerical, 0-10)
	2: Braden [28] (78.71%)	Braden Index (numerical, 0-23)
	2: Geriatric syndrome [29] (100%)	Geriatric syndrome label (binary, yes/no)
**Social frailty indicators**		
	2: MNA [30] (71.87%)	Mini Nutritional Assessment Index (numerical, 0-30)
	2: TSRI [31] (100%)	Table of Social Risk Indicators (numerical, 0-6)
	2: Barber [32] (81.9%)	Barber Index (numerical, 0-9)
	2: Dependence (100%)	Dependence label (binary, yes/no)
**Unhealthy lifestyle habits**		
	2: BMI (74.19%)	BMI (numerical)
	2: Physical activity (75.61%)	Patient’s physical activity (categorical, 3 levels)
	2: Alcohol intake (73.94%)	Patient’s alcohol intake (categorical, 3 levels)
	2: Smoking (73.03%)	Patient’s smoking habits (categorical, 3 levels)
**Use of health care resources**		
	3: Hospital admissions (100%)	Number of admissions during the previous 12 months (numerical)
	3: Emergency room visits (100%)	Number of emergency room visits during the previous 12 months (numerical)
	3: Primary care encounters (100%)	Number of encounters with primary care professionals during the previous 12 months (numerical)
	3: Outpatient visits (100%)	Number of specialized care outpatient visits during the previous 12 months (numerical)
	3: Medication (100%)	Number of drugs prescribed during the previous 12 months (numerical)
	3: Health care expenditure (100%)	Total health care expenses of the previous 12 months in euros (numerical)
**Acute episode complexity**		
	1: Composite Queralt Index [22,23] (100%)	Composite Queralt Index (numerical)
	1: Type of hospitalization (100%)	Type of hospitalization (binary, hospital avoidance/usual care)
	1: Length of stay (100%)	Total hospitalization days (numerical)
	1: Number of active diagnoses (100%)	Number of active diagnoses at admission (numerical)
	1: Leukocyte^a^ (87.33%)	Leukocyte count (numerical)
	1: Lymphocytes^a^ (87.33%)	Percentage of lymphocytes (numerical)
	1: Hemoglobin^a^ (87.33%)	Hemoglobin concentration (numerical)
	1: RDW^a^ (87.33%)	Red blood cell distribution width (numerical)
	1: Glucose^a^ (87.33%)	Glucose concentration (numerical)
	1: Creatinine^a^ (87.33%)	Creatinine concentration (numerical)
	1: Sodium^a^ (87.33%)	Sodium concentration (numerical)
	1: Potassium^a^ (87.33%)	Potassium concentration (numerical)
**Immunization records**		
	2: Vaccination, flu^a^ (100%)	Flu vaccine administration (binary, yes/no)
	2: Vaccination, pneumococcal 13^a^ (100%)	Pneumococcal 13 vaccine administration (binary, yes/no)
	2: Vaccination, pneumococcal 23^a^ (100%)	Pneumococcal 23 vaccine administration (binary, yes/no)

^a^Variables used only for predictive modeling purposes. The number before the variable’s name indicates the database from which the information was retrieved: (1) HCB’s electronic medical records; (2) primary care’s electronic medical records; and (3) the Catalan Health Surveillance System. The percentages of data availability are displayed after variable’s name.

Potential predictors (Table 1) were retrieved from 3 different data sources: (1) HCB’s electronic medical records (EMRs); (2) primary care’s EMR; and (3) the Catalan Health Surveillance System (CHSS) [33]. The latter contains information on clinical diagnoses, medication, and resource utilization from the hospital and primary care. The CHSS is regularly fed from EMR data of all public health care providers in Catalonia paid by CatSalut, the single public payer in Catalonia (ES), which uses it for billing purposes, population-health risk assessment, and allocation of resources. Databases are linked through a unique identification number used for public assurance purposes.

### Ethical Approval

The study was conducted in compliance with the Declaration of Helsinki and was approved by the Ethical Committee for Human Research at the Hospital Clínic of Barcelona (26/04/2017, 2017-0451 and 2017-0452). All data were handled according to the General Data Protection Regulation 2016/679 on data protection and privacy for all individuals within the European Union and the local regulatory framework regarding data protection. Study investigators only had access to a fully anonymized database. Data from other health administrative databases were linked and deidentified by a team not involved in the study analysis.

### Outcomes

The predictive modeling for enhanced patient stratification assessed 2 primary outcomes occurring up to 90 days after discharge: mortality and all-cause hospital readmissions. The clustering analysis allowed the identification and characterization of patients with different risk profiles for personalized service selection purposes. Moreover, the 90-day postdischarge service-utilization trajectories of the identified patients’ risk profiles were analyzed.

### Data Analytics Workflow

From the initial set of 882 patients, 107 were eliminated due to the absence of unrecoverable indispensable data. An additional 14 patients were rejected for subsequent analyses because they died during the hospitalization, resulting in a cohort of 761 patients.

It is to be noted that we observed elevated patterns of missingness in most of the variables recorded in the primary care databases. This was due, in part, to the fact that a vast majority of questionnaires used to assess patient functional characteristics, frailty, and social risks are systematically administered only in elders or patients with explicit evidence of vulnerability or functional decline. Therefore, we imputed baseline levels for Barthel [25], Lawton-Brody [26], Pfeiffer [27], Braden [28], Mini Nutritional Assessment [30], Table of Social Risk Indicators [31], Barber [32] questionnaires in all patients younger than 70 years with no formal diagnosis involving significant levels of dependence, vulnerability, or functional decline. Appendix S1 in Multimedia Appendix 1 presents the diagnostic codes considered for imputation in this initial round (see also [25-32,34]). After that, all variables with percentages of missingness higher than 30% were excluded from the study database. The remaining incomplete registers were imputed using the MissForest [35] algorithm, a robust method for mixed-type data imputation. Furthermore, the categorical features used to encode smoking and alcohol abuse habits were rediscretized to avoid underrepresented categories.

To avoid overfitting, we removed from the study data set all highly correlated features using a Pearson coefficient of 0.75 as a threshold value. In addition, we applied a low variance filtering to remove the features with very few unique values. For this issue, we set the threshold for the ratio between the frequency of the most common value and the frequency of the second most common value to 95:5. The final set of predictors is displayed in Table 1**.**

According to the results of previous predictive modeling experiences in similar settings, reported in Calvo et al [11], we used gradient boosting machines [36] to forecast 2 binary deleterious events occurring up to 90 days after hospital discharge: (1) mortality and (2) all-cause hospital readmissions. We used a grid search to fine-tune the gradient boosting machine parameters (number of trees=1500, maximum number of nodes per tree=5, shrinkage=0.01, and minimum number of observations in terminal nodes=7). The models were trained and tested using a Monte Carlo cross-validation approach with 10 replicates, using 75% of the data for training and the remaining 25% for testing. In addition, every training data partition was 4-fold cross-validated to determine the training and validation splits, accounting for 75% and 25% of the training data partition, respectively. To minimize the effect of class imbalance on the target outcomes caused by the scarcity of unsuccessful cases, we used a random stratified sampling technique [37] to generate the train/test data splits. In addition, to estimate the relative importance of the variables within the predictive models, we performed a mean decrease in accuracy (MDA) analysis. Finally, both predictive models were evaluated, according to the average results of all the independent validations, using the following metrics: area under the receiver operating characteristic curve (AUROC), sensitivity (SE), and specificity (SP). We also calculated the 95% CI for the AUROC using 2000 bootstrapped stratified replicates.

For personalized service selection purposes, we used the K-means clustering algorithm [38,39] to generate groups of patients with similar clinical and social risk profiles. We used the average silhouette method [40] to determine the optimal number of clusters. Finally, the baseline characteristics and patient service utilization trajectories up to 90 days after hospital discharge for all risk profiles identified in this process were assessed.

Categorical variables were summarized as absolute values and frequencies, whereas continuous variables were represented by the mean and the SD or the median and interquartile range. ANOVA, together with post hoc pairwise *t* test (unpaired, 2-tailed), and Kruskal-Wallis, together with post hoc pairwise Wilcoxon tests, were used to assess changes in numeric outcomes, as needed. The Fisher exact test was used to assess changes in categorical variables. Bonferroni adjustments were used in multiple pairwise comparisons. The threshold for statistical significance was set at .05. In addition, to enable multidimensional data combination and to enhance risk profiles (ie, clusters) comparison and visualization, all features were rescaled into a 0-1 range using a minimum-maximum normalization approach. Afterward, all features were aggregated, averaged, and displayed in radar plots in 7 categories that mimic the groups of aforesaid variables, specifically (1) age, (2) medical complexity, (3) functional capacity, (4) social frailty, (5) unhealthy lifestyle habits, (6) use of health care resources, and (7) acute episode complexity.

All the statistical analyses were conducted using R version 4.1.1 [41] (The R Foundation)

## Results

### Characteristics of the Study Population

The average age of the study population was 75.9 (SD 14.51) years. Of the 761 patients overall, 434 (57%) were men and the remaining (n=327, 42.9%) were women. Besides, 63/761 (8.3%) patients died during the following 90 days after hospital discharge, 188/761 (24.7%) had to be readmitted within the study period, and 308/761 (40.5%) had unplanned emergency room (ER) visits. Table 2 presents selected characteristics of the study population, as well as pairwise comparisons between successful and unsuccessful groups: (1) survivors and deceased patients; and (2) patients not requiring hospital readmission and readmitted patients.

In brief, mortality and hospital readmission rates were higher in elders (age: *P*<.001), in highly comorbid and complex patients (AMG: *P*<.001), and in individuals with higher composite Queralt Index (*P*<.001) when combining the severity of the acute episode with the preexisting comorbidity burden. In the entire study group, 77.4% (589/761) of the patients were allocated above the P_95_ of the AMG scoring distribution in Catalonia, the tip of the population-based risk stratification pyramid. The survivors presented a similar distribution (533/698, 76.4%, ≥P_95_), but patients that died after discharge showed a significantly higher (*P*<.001) AMG scoring (56/63, 88.9%, ≥P_95_). Likewise, AMG scoring was markedly lower (*P*<.001) in patients not requiring readmissions (424/573, 74.0%, ≥P_95_) than in those rehospitalized within the 90-day study period (165/188, 87.8%, ≥P_95_). A similar pattern was seen in the composite Queralt Index, reflecting both patient’s complexity and severity of the acute episode (*P*<.001). As expected, total health expenditure at the health system level during the 12 months before the acute episode was also significantly higher in the unsuccessful subgroups than in the entire study group or the successful subsets of patients.

Functional capacity loss (Barthel: *P*<.001) and social frailty and dependence (Barber: *P*<.001; Table S1 in Multimedia Appendix 1) were also identified as potential risk factors for both mortality and hospital readmission. A gender bias was observed in readmitted patients, with men showing a higher hospitalization rate (*P*=.02). A detailed description of all variables included in the analyses is depicted in Table S1 in Multimedia Appendix 1, wherein characteristics of those patients requiring unplanned ER visits during the study period are also displayed.

**Table 2:** Selected traits of the study group depending on mortality and all-cause hospital readmissions.

Variables			All patients (n=761)	Mortality			Readmission		
				Successful (n=698)	Unsuccessful (n=63)	*P* value^a^	Successful (n=573)	Unsuccessful (n=188)	*P* value^a^
**Demographics**									
	Male, n (%)		434 (57.03)	404 (57.88)	30 (47.62)	—	313 (54.62)	121 (64.36)	.02
	Female, n (%)		327 (42.97)	294 (42.12)	33 (52.38)	—	260 (45.38)	67 (35.64)	.02
	Age, mean (SD)		75.06 (14.51)	74.27 (14.53)	83.87 (10.92)	<.001	74.18 (14.87)	77.77 (13.14)	.001
**Medical complexity**									
	AMG^b^ score, mean (SD)		26.35 (14.39)	25.64 (13.97)	34.27 (16.53)	<.001	24.21 (12.95)	32.89 (16.47)	<.001
	**AMG category, n (%)**					<.001			<.001
		Very low risk <P_50_	2 (0.26)	2 (0.29)	0 (0)	—	2 (0.35)	0 (0)	—
		Low risk [P_50_-P_80_)	30 (3.94)	30 (4.3)	0 (0)	—	28 (4.89)	2 (1.06)	.003
		Moderate risk [P_80_-P_95_)	140 (18.4)	133 (19.05)	7 (11.11)	—	119 (20.77)	21 (11.17)	.005
		High risk [P_95_-P_99_)	182 (23.92)	172 (24.64)	10 (15.87)	—	147 (25.65)	35 (18.62)	—
		Very high risk ≥P_99_	407 (53.48)	361 (51.72)	46 (73.02)	<.001	277 (48.34)	130 (69.15)	<.001
**Use of health care resources; 12 months before admission**									
	Health care expenditure in euros^c^, median (IQR)		4164 (2466-7198)	4033 (2418-6930)	5979 (2930-11,072)	<.001	3772 (2260-6343)	5495 (3448-11,235)	<.001
**Acute episode complexity**									
	Length of stay; mean (SD)		7.67 (5.20)	7.42 (4.72)	10.46 (8.49)	.006	7.51 (4.84)	8.11 (6.16)	—
	Composite Queralt Index, mean (SD)		72.73 (30.41)	70.69 (30.02)	95.31 (25.27)	<.001	68.92 (29.75)	84.33 (29.52)	<.001

^a^Only *P* values ≤.05 have been presented.

^b^AMG: Adjusted Morbidity Group.

^c^€1=US $1.08.

### Predictive Modeling

**Figure 1:**
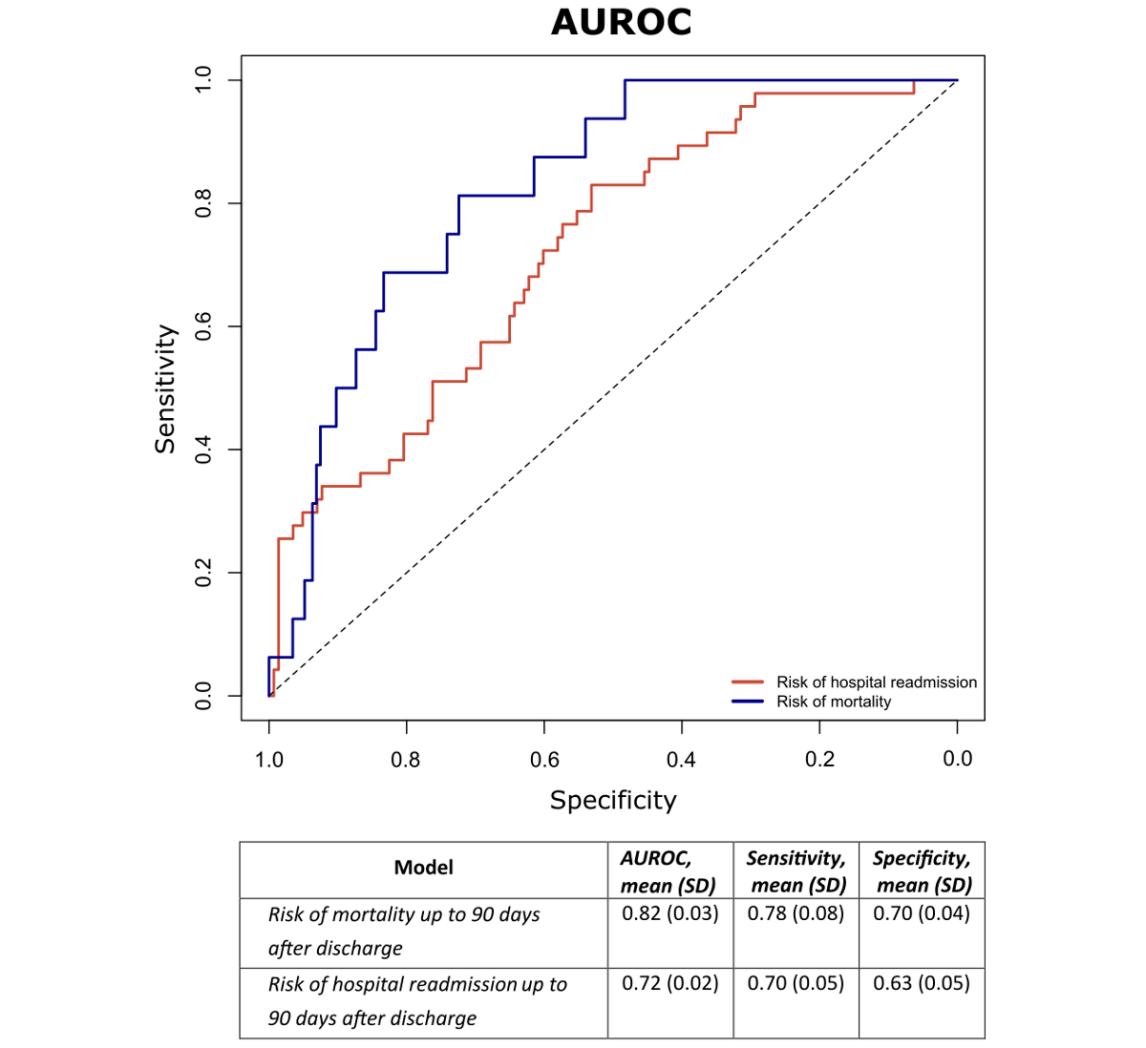
Pooled average receiver operating characteristic curves for both predictive models: risk of mortality is indicated in blue, whereas risk of hospital readmission is indicated in red. The performance of the models is expressed according to the average area under the receiver operating characteristic curves (AUROC), sensitivity, and specificity.

Figure 1 depicts the average performance of the predictive models over the cross-validation process. The mean performance of the models expressed as AUROC (CI; SE/SP) was 0.82 (0.74-0.90; 0.78/0.70) and 0.72 (0.64-0.80; 0.70/0.63) for mortality and all-cause hospital readmission risk, respectively.

Table 3 displays the variable importance weights, according to the MDA analysis, of the 15 most meaningful predictors for both predictive models developed within this study. It is of note that the top 5 predictors for mortality, responsible for 49% accuracy prediction in the MDA analysis, were age (16.7%), composite Queralt Index (12.3%), length of stay (7.7%), Pressure Sore Risk assessed by the Braden scale (6.4%), and heterogeneity of red cell volume/size (6.1%). Overall, variables expressing (1) aging, (2) severity of the acute episode (composite Queralt Index, length of stay, and biological blood markers measured during admission); (3) multimorbidity (number of prescriptions, AMG score, BMI); and (4) frailty (Pfeiffer index) were at the top of the list of the most influential traits modulating mortality after discharge.

Likewise, the top 5 predictors for readmissions during the 90 days after discharge explained 48% accuracy prediction in the MDA analysis. These were the composite Queralt Index (14.8%), blood lymphocytes cell count (10.8%), total health expenditure in the previous year (9.0%), age (8.4%), and AMG score (5.4%). Again, variables associated with the severity of the acute episode (composite Queralt index, peripheral blood biological markers, length of stay), age, multimorbidity (AMG score, health expenditure before the acute episode, BMI, number of specialist outpatient visits), and social frailty (Barber Index) were the main determinants of risk of readmission during the study period.

It should be noted that the predictive role of each of the individual components of the Queralt Index were assessed separately; however, the optimal models’ performance was achieved using Queralt as a composite index.

**Table 3:** The 15 most meaningful predictors for mortality and readmission.

Variable		Variable importance, %
**Predictors for mortality**		
	Age	16.67
	Composite Queralt Index	12.31
	Length of stay	7.68
	Braden Index	6.43
	Red cell distribution width	6.08
	Hemoglobin concentration	4.13
	Number of prescriptions	3.42
	Lymphocytes count	3.31
	Sodium concentration	3.29
	Adjusted Morbidity Group score	3.06
	BMI	2.97
	Potassium concentration	2.95
	Total health care expenditure	2.75
	Glucose concentration	2.40
	Pfeiffer Index	2.25
**Predictors for readmission**		
	Composite Queralt Index	14.77
	Lymphocytes count	10.81
	Total health care expenditure	8.96
	Age	8.42
	Adjusted Morbidity Group score	5.35
	Creatinine concentration	5.33
	BMI	4.30
	Number of primary care visits	4.05
	Hemoglobin concentration	3.92
	Glucose concentration	3.50
	Leukocyte count	3.24
	Number of specialized care visits	3.04
	Red cell distribution width	2.70
	Length of stay	2.53
	Barber Index	2.11

### Patient’s Clustering and Postdischarge Trajectories

**Figure 2:**
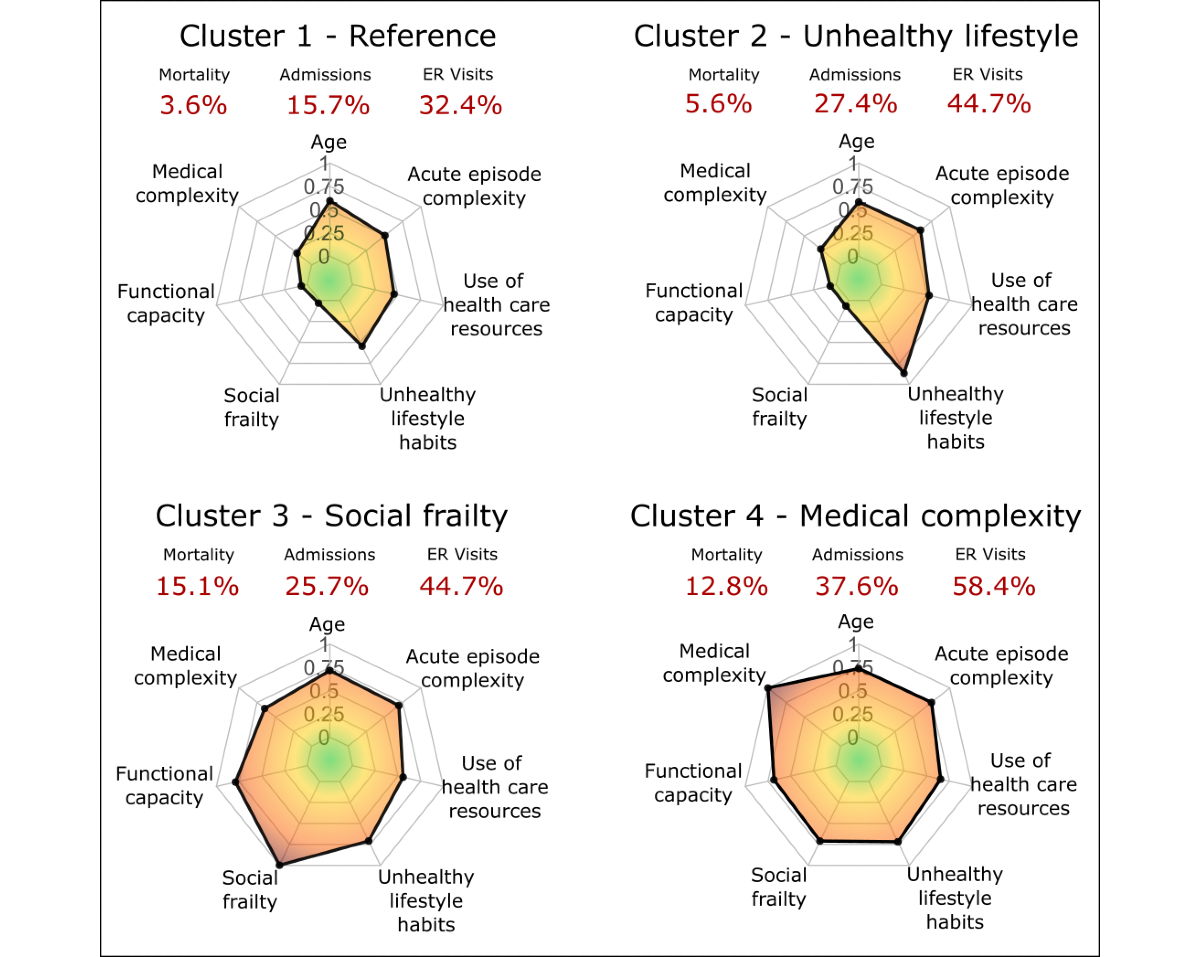
Radar plots of the main characteristics of the 4 clusters. All the features are normalized and grouped into 7 categories: (1) age; (2) medical complexity; (3) functional capacity; (4) social frailty; (5) unhealthy lifestyle habits; (6) use of health care resources; and (7) acute episode complexity. The mortality rates, hospital admissions, and unplanned emergency room visits are displayed in red. ER: emergency room.

We identified 4 relevant clusters of patients whose hallmark characteristics are depicted in Figure 2. The information displayed in the clustering infographics (Figure 2) was normalized and aggregated into scores of 0 to 1 for each of the 7 main dimensions considered in the clustering analysis. The figure also displays mortality rates, hospital admissions, and unplanned ER visits for each cluster during the study period. Each cluster was named according to the most relevant characteristic of the subset of patients: cluster 1 (reference), cluster 2 (unhealthy lifestyle habits), cluster 3 (social frailty), and cluster 4 (medical complexity). An extensive comparison among the 4 clinical groups is displayed in Table S2 in Multimedia Appendix 1.

**Figure 3:**
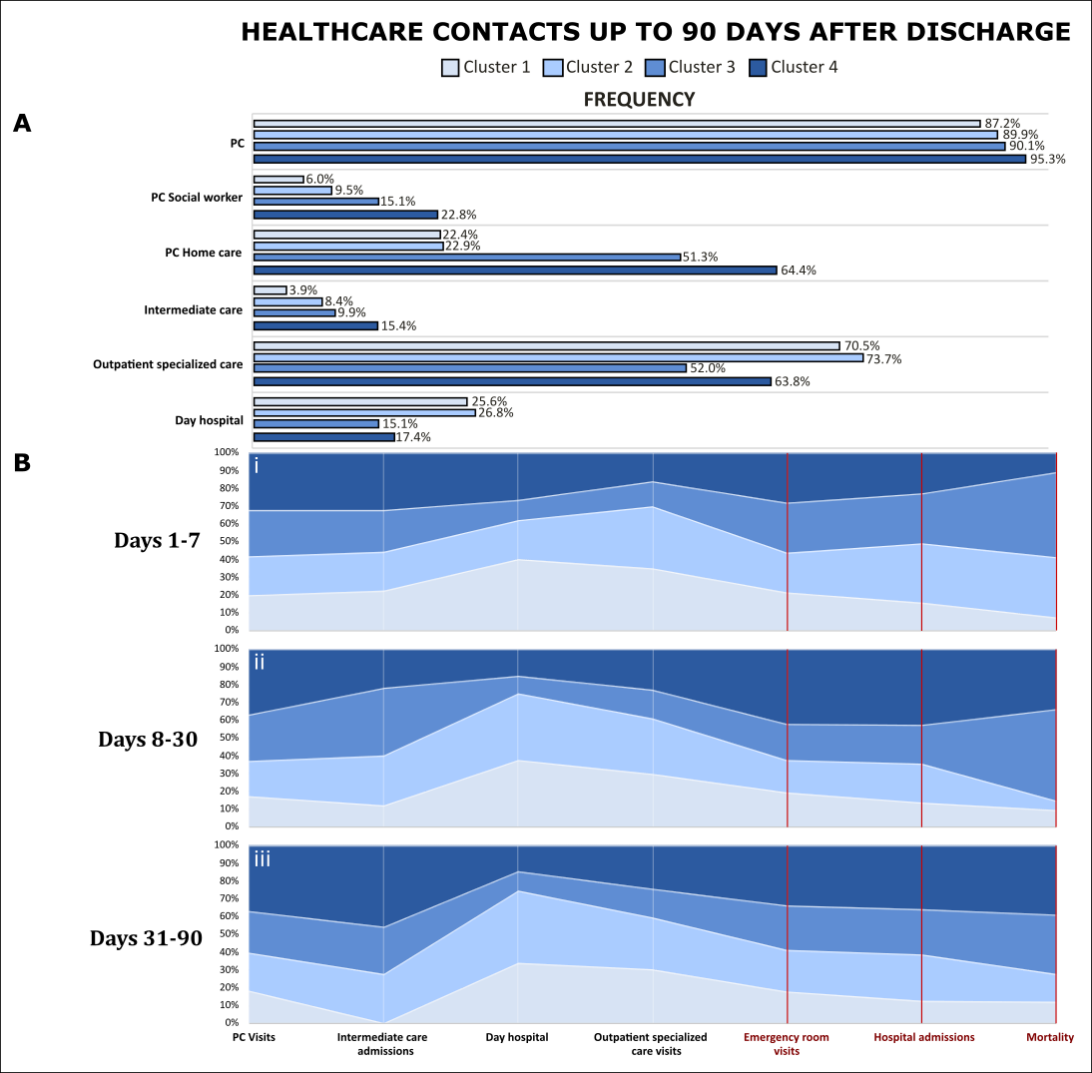
Patients’ trajectories by cluster during the 90-day postdischarge follow-up. (A) Itemized health care contact rates in each cluster. (B) Itemized relative frequencies of the total health care contacts (white) and health outcomes (red) in each cluster assessed in 3 time intervals: (i) days 1-7; (ii) days 8-30; and (iii) days 31-90. PC: primary care.

Figure 3 displays the postdischarge trajectories of patients up to 90 days. Figure 3A depicts rates of patient encounters with health care professionals in each cluster at different levels, namely: (1) primary care (physicians/nurses visits, home-based programs, and social workers visits); (2) intermediate care centers; and (3) specialized care (outpatient clinics and day hospitals visits). Figure 3B displays the postdischarge trajectories for each cluster of patients considering 3 consecutive phases: (1) the first week after discharge (panel i); (2) the 3 subsequent weeks (panel ii); and (3) the last 2 months of the study period (panel iii). For each panel, the ordinate (y-axis) indicates the relative frequencies of each cluster for the variables shown in the abscissa (x-axis), namely: (1) use of health care resources (primary care visits, intermediate care admissions, and specialized care visits), and (2) main outcomes (ER visits, postdischarge hospitalizations, and mortality). Patients’ characteristics of each cluster and the associated postdischarge trajectories are briefly described below. A vast assessment of the health care resources used by the 4 clinical groups up to 90 days after hospital discharge is displayed as follows: (1) rates of patient encounters with health care professionals by cluster (Table S3 in Multimedia Appendix 1); and (2) the total number of contacts with health care professionals by cluster (Table S4 in Multimedia Appendix 1).

The so-called reference patients (cluster 1; 281/761, 36.9%) showed a mean age of 71.0 (SD 15.6) years, with 151/281 (53.7%) being male. The mean AMG scoring was 19.4 (SD 11.0), which corresponds to an elevated morbidity burden, close to the P_95_ of the population-based risk stratification pyramid. Of these, 63/281 (22.4%) patients were included in home care programs targeting complex chronic patients. The average health care expenditure during the previous 12 months of the acute episode was €5491 (US $5952).

Cluster 1 showed the lowest rates of mortality (10/281, 3.6%), readmissions (44/281, 15.7%), and ER visits (91/281, 32.4%) during the 90 days after discharge with no substantial differences among the 3 periods depicted in Figure 3B: (1) the first week, (2) the subsequent 3 weeks, and (3) the last 2 months.

Patients with an unhealthy lifestyle habit profile (cluster 2; 179/179, 23.5%) had a similar age to cluster 1, with a mean of 69.9 (SD 13.4) years; interestingly, 137/179 (76.5%) patients in this cluster were male. The most relevant features in terms of lifestyle were sedentarism, tobacco smoking, and alcohol abuse (Table S2 in Multimedia Appendix 1). The mean AMG scoring was 23.7 (SD 11.7), close to P_97_ of the risk stratification pyramid. The number of patients included in home-based care programs due to complex chronic conditions was 41/179 (22.9%), a figure close to that seen in cluster 1. The average baseline health care expenditure was €6037 (US $6544).

Patients in cluster 2 patients presented a slightly higher mortality rate (10/179, 5.6%), but remarkably higher rates of readmissions (49/179, 27.4%) and ER consultations (80/179, 44.7%) than the reference subset. It is of note, however, that their age, level of functional and social frailty, as well as medical complexity did not show differences with cluster 1. Most importantly, this subset of patients showed a high rate of early mortality during the first week (Figure 3B), corresponding to the 4/10 (40%) deceased patients in this cluster (Table S3 in Multimedia Appendix 1). In addition, the rate of readmissions during the follow-up period was slightly higher than that observed in cluster 3 (39/152, 25.7%).

Patients in the social frailty profile (cluster 3; 152/761, 20%) were older than those in the previous clusters, mean age 81.10 (SD 12.67) years, and only 63/152 (41.4%) were male. Their mean AMG score was high, 30.0 (SD 12.7), corresponding to P_98_. A high percentage of the group (78/152, 51.3%) was included in home-based care programs, and their average baseline health care expenditure was €6232 (US $6755). They presented high levels of medical complexity and functional frailty, but the most characteristic feature was the presence of social frailty. Predominant traits of the group were elderly females with medical complexity and high social vulnerability.

The social frailty group showed the highest rate of mortality (23/152, 15.1%), but similar rates of readmissions (39/152, 25.7%) and ER consultations (68/152, 44.7%) than cluster 2. As displayed in Figure 3B, the mortality rate in the group was higher during the first month after discharge as compared with the last 2-month study period.

Finally, the medical complexity profile (cluster 4; 149/761, 19.6%) integrates a higher proportion of elderly patients, mean age of 82.8 (SD 8.7) years, like in cluster 3, but the patients were predominantly male, 83/149 (55.7%). Their mean AMG was higher than that in the other clusters, mean 39.0 (SD 15.6). This group showed the highest percentage of patients undergoing home-based care programs, 96/149 (64.4%). These patients presented marked functional impairment, as well as medical complexity, with poor outcomes in terms of mortality (19/149, 12.8%), readmissions (56/149, 37.6%), and unplanned ER visits (87/149, 58.4%). Both mortality and readmission rates increased after the first week (Figure 3B) and remained constant throughout the study. The group showed the highest health care expenditure during the previous year: €8510 (US $9224).

As depicted in Figure 3A, the 4 clusters presented high rates of primary care visits with minor differences among them. Clusters 3 and 4 clearly showed the highest use of community-based resources (ie, home-based care programs and visits to social workers and intermediate care), whereas clusters 1 and 2 presented a higher use of specialized care resources, outpatient visits, and day hospital visits than the two other clusters.

## Discussion

### Principal Findings

This study had a 2-fold aim: (1) to assess the risk of mortality and readmission during 90 days after discharge from a tertiary hospital, and (2) to characterize patients’ profiles and their postdischarge trajectories during the study period. The primary purpose of the research was to enhance transitional care after discharge, considering both patients’ risk level and the specificities of their profiles by assessing different dimensions. A careful analysis discarded any impact on the study results associated with patients’ entry point, hospital avoidance program, or conventional hospitalization [17]. Notably, 77.4% (589/761) of the overall study group fell into the top 5% of the regional population-based risk stratification pyramid built-up using the AMG scoring distribution.

### Predictive Modeling

According to the state-of-the-art results [9-12], the proposed machine learning strategy used for computational modeling was adequate to achieve acceptable performance of the predictive models assessing mortality and readmission risks during the study period. The study offers a promising scenario for the future use of computational modeling to feed clinical decision support systems. In addition, the results of this research may guide health professionals in refining personalized transitional care strategies with an integrated care approach, fostering vertical integration between specialized and community-based care, and health and social care.

The results indicate that the most relevant predictors fell into the following 5 categories: (1) age, (2) severity of the acute episode, (3) multimorbidity and complexity, (4) functional, and (5) social frailty. Such a pattern of predictors is fully aligned with a previous report [11] on the predictive modeling of patients undergoing the Hospital at Home (HaH) program at HCB between 2011 and 2015. The statistical analysis in this study suggested synergies between the complexity of the baseline patient’s condition (ie, AMG score) and the severity of the acute episode (ie, composite Queralt Index) leading to increased risk of postdischarge deleterious events. Accordingly, the 2 indices, AMG and Queralt Index, should be included as covariates in the predictions. Moreover, the MDA analysis of the predictive models indicated that different individual variables might play a significant predictive role in the modeling despite having possible weak collinearities.

### Cluster Analysis

The purpose of the cluster analysis was to contribute to defining transitional care pathways fitting the requirements of the identified subsets of patients. In this regard, it seems reasonable to assume that cluster 1, the reference profile, includes candidates for standard patient-centered transitional care. Moreover, this study allowed identifying 2 different care scenarios that are described below.

Patients included in cluster 2, unhealthy lifestyle habits, appear as candidates for preventive strategies that promote healthy lifestyles, including target-oriented cognitive behavioral therapies. Such interventions should be initiated or intensified during the acute episode and continued at the community level with an appropriate follow-up. It is of note that patients within this cluster were predominantly men, with no significant differences in terms of age and medical/social baseline conditions, or severity of the acute episode, as compared with the reference profile. The major distinctive traits were actionable factors, predominantly tobacco smoking and sedentarism but sometimes also alcohol addiction. It should be highlighted that these patients show potentially avoidable high mortality rates during the first week after discharge and potentially avoidable high rates of ER consultations and readmissions across the entire study period.

Clusters 3 (social frailty) and 4 (medical complexity) define a different scenario with common requirements and cluster-specific needs. The 2 subsets include elderly patients, on average 11 years older than clusters 1 and 2, with higher AMG scoring (≥P_98_). Typical recommendations for these 2 clusters are to focus on care-oriented interventions rather than cure and optimizing home-based services to prevent unplanned ER visits and readmissions. To our understanding, clusters 3 and 4 define an ideal scenario for productive interactions among HaH resources, intermediate care, home-based primary care programs, and social support resources. While patients in cluster 3 deserve specific actions to solve social requirements, interventions in cluster 4 should combine addressing complex medical needs, attention to the social context, and providing care based on people’s multidimensional needs.

### Strengths and Limitations

This study shows strengths that provide some uniqueness to the analysis. The articulation of the different data sets described in the “Methods” section represented a significant logistic effort to generate multilevel predictive modeling encompassing different key dimensions that ensured a comprehensive patient characterization. Moreover, the study design used all patients’ information across the health system during 3 successive periods, namely, (1) the entire year before the admission; (2) the acute episode triggering hospitalization; and (3) the 90 days after discharge, which provided the basis for the 2-step protocol using robust statistical tools that were used in this study. Overall, the predictive modeling approach adopted in this study has an exploratory nature while reinforcing conclusions regarding the main determinants of patient outcomes after hospitalization from our prior studies [11].

However, we also acknowledge 2 main study limitations. First, by design, the research was performed on a relatively narrow segment of patients close to the tip of the population-based risk stratification pyramid. Second, the size of the entire study group and the 4 clusters contained a limited number of patients, which may weaken some of the conclusions.

While accepting that the research represents a valuable contribution toward risk stratification of transitional care, we acknowledge that additional studies will be needed to validate the predictive modeling in larger independent populations. Future implementation research should be planned to transform computational modeling into decision support tools to be sustainably adopted, and dynamically updated, into clinical workstations for routine use across health care tiers.

### Value-Generating Strategies for the Management of Multimorbidity

#### Overview

Despite the aforementioned limitations, our report provides highly valuable information and messages that support well-defined strategies leading to enhanced management of multimorbidity in an integrated care scenario showing a clear potential for value generation. We have identified, however, some challenges, at least at 3 different layers.

#### Enhanced Transitional Care After Hospital Discharge

As mentioned earlier, this study has an exploratory nature. The results obtained should require further testing and validation using a large independent study group. Such a study is currently ongoing using a large data set from Catalonia (ES) that includes more than 100,000 patients discharged from different providers following a similar study design. The primary aim of the initiative [42] is to assess the impact and site transferability of HaH. Still, it will also allow validation of the lessons learnt in this study, and it should be the basis for future initiatives that aim to test the recommendations for the different clusters of patients identified in this report.

#### Generating Decision Support Tools for Clinicians

This study aimed to generate decision support tools for clinicians that foster vertical and horizontal integration with a collaborative adaptive case management approach [43]. However, the transfer of the potential of predictive computational modeling approaches, such as the one reported in this research, into decision support tools integrated into clinicians’ workstations constitutes a major challenge involving several levels of complexity, namely, (1) use of appropriate predictors ensuring their availability; (2) testing and continuously assessing clinical decision support systems; and (3) design of user-friendly and properly profiled user interfaces. However, we note that recent digitalization initiatives suited for integrated care scenarios [44] may provide relevant novel contributions to the field.

#### The Generalization of the Approach to Other Use Cases

The current strategy for enhanced transitional care after discharge can be reasonably transferred to the prevention of acute episodes of exacerbation leading to unplanned hospitalizations in high-risk chronic patients. Previous reports have shown the efficacy of preventive interventions [45], as well as proven the need for proper stratification and workforce preparation to generate effectiveness in real-life settings [8]. Our results clearly cover some of the identified needs. However, the most promising scenario is the use of multilevel computational modeling [11,18] for early prediction of target clusters of comorbid conditions (ie, cardiovascular, chronic obstructive pulmonary disease, type 2 diabetes) in susceptible patients. This approach should contribute to the deployment and sustainable adoption of preventive strategies for the management of chronic patients aiming at delaying, or even stopping, their progress toward the tip of the risk stratification pyramid [46,47].

### Conclusions

This study combines multilevel predictive modeling and cluster analysis in a population of comprehensively characterized complex chronic patients discharged from a university hospital. The results indicated the potential to predict mortality and morbidity-related adverse events leading to unplanned hospital readmissions. The resulting patient profiles fostered recommendations for personalized service selection with the capacity for value generation. The lessons learnt show a promising scenario for generating clinical decision support tools for clinicians, enabling value generation within an integrated care scenario involving vertical and horizontal integration.

## Appendix

Multimedia Appendix 1 Diagnostic codes, baseline characteristics of the clinical groups, and rates of patient encounter with health care professionals.

## Abbreviations

AMG: Adjusted Morbidity Groups
AUROC: area under the receiver operating characteristic curve
CHSS: Catalan Health Surveillance System
EMR: electronic medical record
ER: emergency room
HaH: Hospital at Home
HCB: Hospital Clínic of Barcelona
MDA: mean decrease in accuracy
MNA: Mini Nutritional Assessment
SE: sensitivity
SP: specificity
TSRI: Table of Social Risk Indicators

## Data Availability

Data for this study are not publicly available due to patient privacy concerns. The scripts used in this study are available from the corresponding author upon reasonable request.
