# Supplementary material for: Prevention of Unplanned Hospital Admissions in Multimorbid Patients Using Computational Modeling: Observational Retrospective Cohort Study
Source: J Med Internet Res. 2023 Feb 16;25:e40846. doi: 10.2196/40846 (PMC9982720; doi:10.2196/40846)
Supplement: Multimedia Appendix 1 [file jmir_v25i1e40846_app1.docx]

**COMPUTATIONAL MODELLING FOR PREVENTION OF UNPLANNED HOSPITAL ADMISSIONS IN MULTIMORBID PATIENTS**

Rubèn González-Colom et al.

*(On-line supplementary material)*

The current on-line supplementary material provides detailed information on the methodological approach adopted in the current study, as well as additional data complementing the description of the results showed in the main manuscript.

# METHODS

During the data preparation phase, we observed elevated patterns of missingness in most of the variables recorded in the primary care databases. This is in part because a vast majority of questionnaires employed to assess patient functional characteristics, frailty and social risks are systematically performed only in elders or patients with some evidence of vulnerability and/or functional decline.

Therefore, we imputed basal levels for Barthel^1^, Lawton-Brody^2^, Pfeiffer^3^, Braden^4^, Mini Nutritional Assessment (MNA)^5^, Table of Social Risk Indicators (TIRS)^6^, Barber^7^ questionnaires in all patients younger than 70 years with no formal diagnosis of:

1) **Palliative care:** Z51.5

2) **Dependence:** Z63.6, Z63.9, Z73.6, Z54.9

3) **Geriatric syndrome**^8^ **(at least 3 diagnostic codes from the following list):** Z60.02, R63.4, E43, E44.1, E44.0. E46, R53, Z63.6, Z73.6, Z54,9, F03.90, F01.50, G30.90, G31.83, F06.7, R41.3, T14.8XXA, L89.90, L97.909, I70.25, I83.009, W19.9, R32, R15, F98.1, N39.3, N39.41, N39.46, N39.490, N39.498, F05, H53, H54, H90, H91, H93.

* All the diagnostic codes are in ICD-10-CM^9^

# RESULTS

**Table 1S** depicts the baseline characteristics of the study population itemized in eight domains of data: i) sociodemographic information; ii) population-based registry indicators on morbidity and complexity; iii) patients’ functional characteristics; iv) frailty and social risk indicators; v) unhealthy lifestyle habits; vi) utilization of healthcare resources; vii) clinical and biological data collected during the acute episode; and, viii) immunization records. **Table 1S** also shows pairwise comparisons between successful and unsuccessful groups; that is: i) survivors and deceased patients; ii) patients not requiring hospital readmission and readmitted patients; and, iii) patients not requiring unplanned emergency room visits and patients visited in the hospital emergency room service.

**Table 2S** describes the baseline characteristics of the four clinical groups identified in the clustering analysis itemized in seven domains of data: i) sociodemographic information; ii) population-based registry indicators on morbidity and complexity; iii) patients’ functional characteristics; iv) frailty and social risk indicators; v) unhealthy lifestyle habits; vi) utilization of healthcare resources; and, vii) clinical and biological data collected during the acute episode. The table also displays rates of mortality, hospital admissions and unplanned emergency room visits for each cluster during the study period.

The patients’ post-discharge trajectories during the study period are displayed in **Tables 3-4S.** Concretely, **Table 3S** depicts the rates of patient’s encounters with healthcare professionals in clinical group at different healthcare levels, namely: i) primary care (Physicians/Nurses visits, Home-based programs, and Social workers visits); ii) intermediate care centres; and iii) specialized care (outpatient clinics and day hospitals visits). In addition, **Table 4S** displays the total number of healthcare contacts. In both tables, the study period has been fragmentated in three temporal frames of interest, explicitly: i) first week after discharge; ii) three subsequent weeks; and iii) last two months of the study period. The tables also display rates of mortality, hospital admissions and unplanned emergency room visits.

**Table 1S –** *Baseline characteristics of the study group depending upon mortality, all-cause hospital readmissions and unplanned emergency room visits. All the values are accordingly expressed as n(%) or mean(sd). Only the p-values ≤ 0.05 have been displayed.*

| **Variables** | **All patients** | **Mortality** | | | **Readmission** | | | **Emergency room** | | |
| --- | --- | --- | --- | --- | --- | --- | --- | --- | --- | --- |
|  |  | **Succesful** | **Unsuccesful** | **p-value** | **Succesful** | **Unsuccesful** | **p-value** | **Succesful** | **Unsuccesful** | **p-value** |
|  | **n=761** | **n=698 (91.72%)** | **n=63 (8.28%)** |  | **n=573 (75.30%)** | **n=188 (24.70%)** |  | **n=453 (59.53 %)** | **n=308 (40.47 %)** |  |
| **Demographics** | | | | | | | | | | |
| *Male; n(%)* | 434 (57.03) | 404 (57.88) | 30 (47.62) |  | 313 (54.62) | 121 (64.36) | .021 | 263 (58.05) | 171 (55.52) |  |
| *Female; n(%)* | 327 (42.97) | 294 (42.12) | 33 (52.38) |  | 260 (45.38) | 67 (35.64) |  | 190 (41.95) | 137 (44.48) |  |
| *Age; mean(sd)* | 75.06 (14.51) | 74.27 (14.53) | 83.87 (10.92) | <.001 | 74.18 (14.87) | 77.77 (13.14) | .001 | 73.91 (14.43) | 76.76 (14.47) | .007 |
| **Medical complexity** | | | | | | | | | | |
| *AMG score; mean(sd)* | 26.35 (14.39) | 25.64 (13.97) | 34.27 (16.53) | <.001 | 24.21 (12.95) | 32.89 (16.47) | <.001 | 23.22 (12.73) | 30.96 (15.42) | <.001 |
| *AMG category, n (%)* |  |  |  |  |  |  |  |  |  |  |
| *Very low risk < P_50_* | 2 (0.26) | 2 (0.29) | 0 (0) | <.001 | 2 (0.35) | 0 (0) | <.001 | 1 (0.22) | 1 (0.32) | <.001 |
| *Low risk [P_50_ - P_80_)* | 30 (3.94) | 30 (4.3) | 0 (0) |  | 28 (4.89) | 2 (1.06) |  | 24 (5.3) | 6 (1.95) |  |
| *Moderate risk [P_80_-P_95_)* | 140 (18.4) | 133 (19.05) | 7 (11.11) |  | 119 (20.77) | 21 (11.17) |  | 103 (22.74) | 37 (12.01) |  |
| *High risk [P_95_-P_99_)* | 182 (23.92) | 172 (24.64) | 10 (15.87) |  | 147 (25.65) | 35 (18.62) |  | 124 (27.37) | 58 (18.83) |  |
| *Very high risk ≥ P_99_* | 407 (53.48) | 361 (51.72) | 46 (73.02) |  | 277 (48.34) | 130 (69.15) |  | 201 (44.37) | 206 (66.88) |  |
| *CCP; n(%)* | 164 (21.55) | 139 (19.91) | 25 (39.68) | <.001 | 102 (17.80) | 62 (32.98) | <.001 | 65 (14.35) | 99 (32.14) | <.001 |
| *ACP; n(%)* | 15 (1.97) | 10 (1.43) | 5 (7.93) | .005 | 12 (2.09) | 3 (1.59) |  | 9 (1.99) | 6 (1.94) |  |
| *PIIC; n(%)* | 209 (27.46) | 175 (25.07) | 34 (53.96) | <.001 | 135 (23.56) | 74 (39.36) | <.001 | 92 (20.31) | 117 (37.98) | <.001 |
| **Patient's functional capacity** | | | | | | | | | | |
| *Barthel [0-100]; median(IQR)* | 100 (76 - 100) | 100 (80 -100) | 75 (43 - 100) | <.001 | 100 (80 -100) | 100 (69 - 100) | .002 | 100 (80 -100) | 100 (70 - 100) | <.001 |
| *Lawton-Brody [0-8]; median(IQR)* | 8 (5 - 8) | 8 (5 - 8) | 5 (4 - 8) | <.001 | 8 (5 - 8) | 8 (4 - 8) | <.001 | 8 (6 - 8) | 8 (4 - 8) | <.001 |
| *Pfeiffer [0-10]; median(IQR)* | 0 (0 - 1) | 0 (0 - 1) | 2 (0 - 5) | <.001 | 0 (0 - 1) | 0 (0 - 2) | <.001 | 0 (0 - 1) | 0 (0 -2) | <.001 |
| *Braden[0-23]; median(IQR)* | 23 (19 -23) | 23 (20 - 23) | 20 (16 -23) | <.001 | 23 (20 - 23) | 22 (18 -23) | <.001 | 23 (20 - 23) | 22 (18 - 23) | <.001 |
| *Geriatric syndrome; n(%)* | 87 (11.43) | 77 (11.03) | 10 (15.87) |  | 69 (12.04) | 18 (9.57) |  | 49 (10.82) | 38 (12.38) |  |
| **Social frailty indicators** | | | | | | | | | | |
| *MNA [0-30]; median(IQR)* | 30 (29 - 30) | 30 (29 - 30) | 29 (28 - 30) | <.001 | 30 (29 - 30) | 30 (29 - 30) | .007 | 30 (30 - 30) | 30 (29 -30) | <.001 |
| *TIRS [0-9] >0 ; n(%)* | 79 (10.38) | 70 (10.03) | 9 (14.29) |  | 50 (8.73) | 29 (15.43) |  | 36 (7.95) | 43 (13.96) |  |
| *Barber [0-9] > 0; n(%)* | 242 (31.80) | 207 (29.66) | 35 (55.56) | <.001 | 170 (29.67) | 72 (38.30) | <.001 | 124 (27.37) | 118 (38.31) | <.001 |
| *Dependence; n(%)* | 146 (19.19) | 121 (17.34) | 25 (39.68) | <.001 | 100 (17.45) | 46 (24.46) | .042 | 71 (15.67) | 75 (24.35) | .003 |
| **Unhealty lifestyle habits** | | | | | | | | | | |
| *Body Mass Index; mean (sd)* | 28.33 (4.86) | 28.48 (4.92) | 26.52 (3.60) | <.001 | 28.48 (4.89) | 27.83 (4.72) |  | 28.40 (4.74) | 28.20 (5.02) |  |
| *Physical activity; n(%)* |  |  |  |  |  |  |  |  |  |  |
| *Active* | 481 (63.20) | 451 (64.61) | 30 (47.62) | .002 | 383 (66.84) | 98 (52.13) | <.001 | 316 (69.76) | 165 (53.57) | <.001 |
| *Insufficiently active* | 143 (18.80) | 132 (18.91) | 11 (17.46) |  | 97 (16.93) | 46 (23.40) |  | 72 (15.89) | 71 (23.05) |  |
| *Sedentary* | 137 (18.00) | 115 (16.48) | 22 (34.92) |  | 93 (16.23) | 44 (24.47) |  | 65 (14.35) | 72 (27.38) |  |
| *Alcohol intake; n(%)* |  |  |  |  |  |  |  |  |  |  |
| *non-drinker* | 438 (57.55) | 390 (55.87) | 48 (76.19) | .012 | 323 (56.37) | 115 (61.17) |  | 241 (53.20) | 197 (63.96) | .010 |
| *low risk* | 318 (41.79) | 303 (43.41) | 15 (23.81) |  | 246 (42.94) | 72 (38.30) |  | 208 (45.92) | 110 (35.72) |  |
| *high risk* | 5 (0.66) | 5 (0.72) | 0 (0) |  | 4 (0.69) | 1 (0.53) |  | 4 (0.88) | 1 (0.32) |  |
| *Smoking; n(%)* |  |  |  |  |  |  |  |  |  |  |
| *current smoker* | 92 (12.09) | 90 (12.90) | 2 (3.17) |  | 70 (12.22) | 22 (11.70) |  | 60 (13.25) | 32 (10.39) |  |
| *former smoker* | 122 (16.03) | 112 (16.04) | 10 (15.88) |  | 84 (14.66) | 38 (20.22) |  | 75 (16.55) | 47 (15.26) |  |
| *non-smoker* | 547 (71.88) | 496 (71.06) | 51 (80.95) |  | 419 (73.12) | 128 (68.08) |  | 318 (70.20) | 229 (74.35) |  |
| **Use of healthcare resources; 12m pre-admission** | | | | | | | | | | |
| *Total healthcare expenditure; median(IQR)* | 4,164 (2,466 - 7,198) | 4,033 (2,418 - 6,930) | 5,979 (2,930 - 11,072) | <.001 | 3,772 (2,260 - 6,343) | 5,495 (3,448 - 11,235) | <.001 | 3,517 (2,094 - 6,113) | 4,886 (3,281 - 9,427) | <.001 |
| *Number of prescriptions; mean(sd)* | 8.17 (4.36) | 8.03 (4.34) | 9.74 (4.34) | <.001 | 7.7 (4.06) | 9.61 (4.90) | <.001 | 7.47 (4.04) | 9.21 (4.60) | <.001 |
| *Primary care visits; median(IQR)* | 14 (8 - 25) | 13 (8 - 24) | 16 (10 - 28) | <.001 | 12 (7 -21) | 18 (11 - 30) | <.001 | 12 (7 - 19) | 17 (10 -30) | <.001 |
| *Outpatient visits; median(IQR)* | 3 (1 - 7) | 3 (1 - 7) | 2 (1 - 8) |  | 3 (1 - 6) | 5 (1 - 10) | <.001 | 3 (1 - 6) | 4 (1 - 9) | <.001 |
| *Emergency room visits; median(IQR)* | 2 (1 - 4) | 2 (1 - 4) | 3 (2 - 4) |  | 2 (1 - 3) | 3 (2 - 5) | <.001 | 2 (1 - 3) | 3 (2 -4) | <.001 |
| *Hospital admissions; median(IQR)* | 1 (0 - 1) | 1 (0 - 1) | 1 (1 - 2) | .047 | 1 (0 - 1) | 1 (1 - 2) | <.001 | 1 (0 - 1) | 1 (0 - 2) | <.001 |
| **Acute episode complexity** | | | | | | | | | | |
| *Number of active diagnoses; mean(sd)* | 17.96 (9.05) | 17.84 (9.17) | 19.38 (7.69) |  | 17.27 (8.89) | 20.09 (9.27) | <.001 | 16.65 (8.43) | 19.91 (9.59) | <.001 |
| *Length of stay; mean(sd)* | 7.67 (5.20) | 7.42 (4.72) | 10.46 (8.49) | .006 | 7.51 (4.84) | 8.11 (6.16) |  | 7.60 (5.22) | 7.76 (5.17) |  |
| *Composite Queralt index; mean(sd)* | 72.73 (30.41) | 70.69 (30.02) | 95.31 (25.27) | <.001 | 68.92 (29.75) | 84.33 (29.52) | <.001 | 66.90 (29.09) | 81.29 (30.32) | <.001 |
| *Leucocytes count; mean(sd)* | 8.77 (4.37) | 8.72 (4.21) | 9.40 (5.90) |  | 8.68 (4.22) | 9.07 (4.80) |  | 8.60 (4.18) | 9.03 (4.63) |  |
| *Lymphocytes count; mean(sd)* | 17.90 (9.70) | 18.24 (9.89) | 14.17 (6.18) | <.001 | 18.82 (9.7) | 15.12 (9.19) | <.001 | 18.77 (9.51) | 16.63 (9.85) | .003 |
| *Haemoglobin concentration; mean(sd)* | 123.49 (18.08) | 124.20 (18.13) | 115.58 (15.61) | <.001 | 124.40 (18.46) | 120.72 (16.62) | .011 | 124.32 (18.39) | 122.26 (17.57) |  |
| *Red cell distribution width; mean(sd)* | 14.84 (1.64) | 14.78 (1.63) | 15.55 (1.71) | <.001 | 14.70 (1.65) | 15.25 (1.57) | <.001 | 14.60 (1.51) | 15.19 (1.77) | <.001 |
| *Glucose concentration; mean(sd)* | 127.94 (57.67) | 127.74 (57.74) | 130.14 (57.36) |  | 123.96 (51.94) | 140.09 (71.18) | .004 | 123.04 (52.79) | 135.15 (63.58) | .003 |
| *Creatinine concentration; mean(sd)* | 1.10 (0.62) | 1.09 (0.59) | 1.27 (0.88) |  | 1.06 (0.59) | 1.22 (0.71) | .007 | 1.08 (0.60) | 1.13 (0.65) |  |
| *Sodium concentration; mean(sd)* | 140.58 (3.65) | 140.61 (3.60) | 140.26 (4.24) |  | 140.57 (3.55) | 140.62 (3.97) |  | 140.52 (3.40) | 140.68 (4.01) |  |
| *Potassium concentration; mean(sd)* | 4.11 (0.54) | 4.11 (0.53) | 4.20 (0.62) |  | 4.09 (0.51) | 4.20 (0.61) | .019 | 4.09 (0.51) | 4.14 (0.58) |  |
| **Inmmunization records** | | | | | | | | | | |
| *Flu; n(%)* | 520 (68.33) | 469 (67.19) | 51 (80.95) | .024 | 382 (66.66) | 138 (73.40) |  | 299 (66.00) | 221 (71.75) |  |
| *Pneumococcal 13; n(%)* | 26 (3.42) | 25 (3.58) | 1 (1.58) |  | 21 (3.66) | 5 (2.65) |  | 15 (3.31) | 11 (3.57) |  |
| *Pneumococcal 23; n(%)* | 475 (62.42) | 427 (61.17) | 48 (76.19) | .02 | 340 (59.34) | 135 (71.80) | .002 | 270 (59.60) | 205 (66.55) |  |

**Table 2S –** *Baseline characteristics of the four clinical groups, namely: Cluster 1: Reference patients; Cluster 2: Unhealthy lifestyle; Cluster 3: Frailty; Cluster 4: Medical complexity. All the values are accordingly expressed as n(%) or mean(sd). Only the p-values ≤ 0.05 have been displayed.*

|  | **Cluster 1** | **Cluster 2** | **Cluster 3** | **Cluster 4** | **P-value** |
| --- | --- | --- | --- | --- | --- |
|  | **n=281 (36.93%)** | **n=179 (23.52%)** | **n=152 (19.97%)** | **n=149 (19.58%)** |  |
| **Sociodemographic data** | | | | | |
| *Men, n(%)* | 151 (53.74) | 137 (76.54) | 63 (41.45) | 83 (55.7) | <.001 |
| *Women, n(%)* | 130 (46.26) | 42 (23.46) | 89 (58.55) | 66 (44.3) | <.001 |
| *Age, mean(sd)* | 70.99 (15.63) | 69.91 (13.44) | 81.06 (12.67) | 82.84 (8.74) | <.001 |
| **Medical Complexity** | | | | | |
| *AMG score, mean(sd)* | 19.41 (11.06) | 23.72 (11.69) | 29.95 (12.09) | 38.96 (15.64) | <.001 |
| *AMG category, n (%)* |  |  |  |  |  |
| *Very low risk < P_50_* | 1 (0.36) | 1 (0.56) | 0 (0) | 0 (0) | <.001 |
| *Low risk [P_50_ - P_80_)* | 21 (7.47) | 8 (4.47) | 1 (0.66) | 0 (0) |  |
| *Moderate risk [P_80_-P_95_)* | 87 (30.96) | 35 (19.55) | 13 (8.55) | 5 (3.36) |  |
| *High risk [P_95_-P_99_)* | 86 (30.6) | 46 (25.7) | 33 (21.71) | 17 (11.41) |  |
| *Very high risk ≥ P_99_* | 86 (30.6) | 89 (49.72) | 105 (69.08) | 127 (85.23) |  |
| *CCP, n(%)* | 2 (0.71) | 5 (2.79) | 28 (18.42) | 129 (86.58) | <.001 |
| *ACP, n(%)* | 0 (0) | 3 (1.68) | 6 (3.95) | 6 (4.03) | .001 |
| *PIIC, n(%)* | 5 (1.78) | 3 (1.68) | 55 (36.18) | 146 (97.99) | <0.001 |
| **Patient's functional capacity** | | | | | |
| *Lawton Brody, mean(sd)* | 7.91 (0.6) | 7.87 (0.63) | 4.26 (1.91) | 4.95 (2.69) | <.001 |
| *Pfeiffer, mean(sd)* | 0.25 (1.18) | 0.06 (0.35) | 2.91 (2.94) | 1.93 (2.69) | <.001 |
| *Braden, mean(sd)* | 22.63 (1.48) | 22.65 (1.01) | 17.95 (2.91) | 18.85 (3.38) | <.001 |
| *Geriatric syndrome, n(%)* | 4 (1.42) | 5 (2.79) | 44 (28.95) | 34 (22.82) | <.001 |
| **Social frailty indicators** | | | | | |
| *MNA, mean(sd)* | 29.96 (0.2) | 29.96 (0.19) | 28.32 (2.27) | 29.02 (2.77) | <.001 |
| *TIRS >0, n(%)* | 4 (1.42) | 4 (2.23) | 35 (23.03) | 36 (24.16) | <.001 |
| *Barber >0, n(%)* | 5 (1.78) | 7 (3.91) | 150 (98.68) | 80 (53.69) | <.001 |
| *Dependency, n(%)* | 0 (0) | 1 (0.56) | 123 (80.92) | 22 (14.77) | <.001 |
| **Unhealthy lifestyle habits** | | | | | |
| *BMI, mean(sd)* | 28.56 (5.11) | 28.12 (4.28) | 27.94 (4.71) | 28.42 (5.16) |  |
| *Physical activity; n(%)* |  |  |  |  |  |
| *Active* | 225 (80.07) | 130 (72.63) | 59 (38.82) | 67 (44.97) | <.001 |
| *Insufficiently active* | 40 (14.23) | 44 (24.58) | 32 (21.05) | 28 (18.79) |  |
| *Sedentary* | 16 (5.69) | 5 (2.79) | 61 (40.13) | 54 (36.24) |  |
| *Alcohol intake; n(%)* |  |  |  |  |  |
| *non-drinker* | 144 (51.25) | 53 (29.61) | 127 (83.55) | 113 (75.84) | <.001 |
| *low risk* | 135 (48.04) | 124 (69.27) | 24 (15.79) | 36 (24.16) |  |
| *high risk* | 2 (0.71) | 2 (1.12) | 1 (0.66) | 0 (0) |  |
| *Smoking; n(%)* |  |  |  |  |  |
| *current smoker* | 279 (99.29) | 17 (9.5) | 131 (86.18) | 116 (77.85) | <.001 |
| *former smoker* | 2 (0.71) | 87 (48.6) | 14 (9.21) | 19 (12.75) |  |
| *non-smoker* | 0 (0) | 75 (41.9) | 7 (4.61) | 14 (9.4) |  |
| **Use of healthcare resources; 12m pre-admission** | | | | | |
| *Average healthcare expenditure, mean(sd)* | 5491.36 (7665.35) | 6037.14 (7822.71) | 6231.51 (5989.98) | 8509.83 (7735.83) | <.001 |
| *Number of prescriptions, mean(sd)* | 6.5 (3.55) | 7.72 (4.35) | 9.16 (4.04) | 10.87 (4.53) | <.001 |
| *Number of Primary Care visits, mean(sd)* | 13.39 (16.91) | 15.86 (16.84) | 21.95 (15.67) | 33.74 (29.82) | <.001 |
| *Number of outpatient visits, mean(sd)* | 4.63 (6.21) | 5.93 (6.58) | 3.82 (4.82) | 6.48 (6.84) |  |
| *Number of emergency room visits, mean(sd)* | 2.53 (1.94) | 2.89 (2.21) | 2.93 (2.69) | 3.45 (2.53) | <.001 |
| *Number of hospital admissions, mean(sd)* | 0.8 (0.87) | 0.94 (1.03) | 1.17 (1.38) | 1.32 (1.29) | <.001 |
| **Acute episode complexity** | | | | | |
| *Composite Queralt index, mean(sd)* | 56.22 (24.43) | 69.4 (31.21) | 84.95 (24.38) | 95.38 (25.33) | <.001 |
| *Length of stay, mean(sd)* | 7.11 (4.16) | 7.8 (5.39) | 7.6 (5.29) | 8.62 (6.41) | .009 |
| *Number of active diagnoses mean(sd)* | 13.64 (7.25) | 16.96 (9.05) | 21.82 (8.27) | 23.43 (8.47) | <.001 |
| **Clinical outcomes at 90-days post discharge** | | | | | |
| *Emergency room visits, n(%)* | 91 (32.38) | 62 (34.64) | 68 (44.74) | 87 (58.39) | <.001 |
| *Hospital admissions, n(%)* | 44 (15.66) | 49 (27.37) | 39 (25.66) | 56 (37.58) | <.001 |
| *Mortality, n(%)* | 11 (3.91) | 10 (5.59) | 23 (15.13) | 19 (12.75) | <.001 |

**Table 3S –** *Rates of patient’s encounters with healthcare professionals itemized in four clinical groups, namely: Cluster 1: Reference patients; Cluster 2: Unhealthy lifestyle; Cluster 3: Frailty; Cluster 4: Medical complexity. All the values are accordingly expressed as n(%), standing for: number of patients visited/admitted at least once (relative frequency within the cluster). Only the p-values ≤ 0.05 have been displayed.*

| **Cluster** | **n (%)** | **Primary care  visits** | **p-value** | **Intermediate care  admissions** | **p-value** | **Day hospital  visits** | **p-value** | **Outpatient  specialized care  visits** | **p-value** | **Emergency room  visits** | **p-value** | **Hospital  admissions** | **p-value** | **Mortality** | **p-value** |
| --- | --- | --- | --- | --- | --- | --- | --- | --- | --- | --- | --- | --- | --- | --- | --- |
| *Days 1 -7* | | | | | | | | | | | | | | | |
| 1 | 281 (36.93) | 118 (41.99) | .025 | 9 (3.20) |  | 17 (6.05) |  | 44 (15.66) | .001 | 15 (5.34) |  | 6 (2.14) |  | 1 (0.36) |  |
| 2 | 179 (23.52) | 84 (46.93) |  | 6 (3.35) |  | 7 (3.91) |  | 29 (16.20) |  | 11 (6.15) |  | 10 (5.59) |  | 4 (2.23) |  |
| 3 | 152 (19.97) | 90 (59.21) |  | 5 (3.29) |  | 4 (2.63) |  | 12 (7.89) |  | 15 (9.87) |  | 8 (5.26) |  | 4 (2.63) |  |
| 4 | 149 (19.58) | 95 (63.76) |  | 9 (6.04) |  | 5 (3.36) |  | 15 (10.07) |  | 16 (10.74) |  | 7 (4.70) |  | 1 (0.67) |  |
| *Days 8 - 30* | | | | | | | | | | | | | | | |
| 1 | 280 (37.28) | 155 (55.36) | .002 | 3 (1.07) |  | 43 (15.36) | .002 | 110 (39.29) | <.001 | 35 (12.50) | .01 | 14 (5.00) | .009 | 3 (1.07) | .003 |
| 2 | 175 (23.3) | 111 (63.43) |  | 5 (2.86) |  | 24 (13.71) |  | 81 (46.29) |  | 22 (12.57) |  | 15 (8.57) |  | 1 (0.57) |  |
| 3 | 148 (19.71) | 102 (68.92) |  | 6 (4.05) |  | 9 (6.08) |  | 37 (25.00) |  | 21 (14.19) |  | 12 (8.11) |  | 9 (6.08) |  |
| 4 | 148 (19.71) | 110 (74.32) |  | 4 (2.70) |  | 11 (7.43) |  | 50 (33.78) |  | 37 (25.00) |  | 23 (15.54) |  | 6 (4.05) |  |
| *Days 31 - 90* | | | | | | | | | | | | | | | |
| 1 | 277 (37.84) | 203 (73.29) | .001 | 0 (0.00) | <.001 | 44 (15.88) |  | 160 (57.76) |  | 64 (23.10) | .005 | 27 (9.75) | <.001 | 7 (2.53) | .024 |
| 2 | 174 (23.77) | 143 (82.18) |  | 8 (4.60) |  | 35 (20.11) |  | 101 (58.05) |  | 48 (27.59) |  | 37 (21.26) |  | 5 (2.87) |  |
| 3 | 139 (18.99) | 119 (85.61) |  | 7 (5.04) |  | 18 (12.95) |  | 64 (46.04) |  | 40 (28.78) |  | 27 (19.42) |  | 10 (7.19) |  |
| 4 | 142 (19.4) | 126 (88.73) |  | 15 (10.56) |  | 17 (11.97) |  | 77 (54.23) |  | 58 (40.85) |  | 39 (27.46) |  | 12 (8.45) |  |

**Table 4S –** *Total number of encounters with healthcare professionals itemized in four clinical groups, namely: Cluster 1: Reference patients; Cluster 2: Unhealthy lifestyle; Cluster 3: Frailty; Cluster 4: Medical complexity. All the values are accordingly expressed as n(%), standing for: total number of healthcare contacts (average number of contacts per 100 patients). Only the p-values ≤ 0.05 have been displayed.*

| **Cluster** | **n** | **Primary care  visits** | **p-value** | **Intermediate care  admissions** | **p-value** | **Day hospital  visits** | **p-value** | **Outpatient  specialized care  visits** | **p-value** | **Emergency room  visits** | **p-value** | **Hospital  admissions** | **p-value** |
| --- | --- | --- | --- | --- | --- | --- | --- | --- | --- | --- | --- | --- | --- |
| Days 1 -7 | | | | | | | | | | | | | |
| 1 | 281 (36.93) | 245 (87.19) | <.001 | 9 (3.2) |  | 22 (7.83) |  | 51 (18.15) | .001 | 20 (7.12) |  | 7 (2.49) |  |
| 2 | 179 (23.52) | 179 (100) |  | 6 (3.35) |  | 8 (4.47) |  | 34 (18.99) |  | 14 (7.82) |  | 10 (5.59) |  |
| 3 | 152 (19.97) | 205 (134.87) |  | 6 (3.95) |  | 4 (2.63) |  | 13 (8.55) |  | 17 (11.18) |  | 8 (5.26) |  |
| 4 | 149 (19.58) | 269 (180.54) |  | 9 (6.04) |  | 10 (6.71) |  | 16 (10.74) |  | 18 (12.08) |  | 7 (4.7) |  |
| Days 8 - 30 | | | | | | | | | | | | | |
| 1 | 280 (37.28) | 419 (149.64) | <.001 | 4 (1.43) |  | 61 (21.79) | .002 | 155 (55.36) | <.001 | 41 (14.64) | .003 | 14 (5.00) | .006 |
| 2 | 175 (23.3) | 314 (179.43) |  | 6 (3.43) |  | 40 (22.86) |  | 107 (61.14) |  | 26 (14.86) |  | 15 (8.57) |  |
| 3 | 148 (19.71) | 354 (239.19) |  | 7 (4.73) |  | 9 (6.08) |  | 46 (31.08) |  | 24 (16.22) |  | 13 (8.78) |  |
| 4 | 148 (19.71) | 505 (341.22) |  | 4 (2.70) |  | 14 (9.46) |  | 68 (45.95) |  | 51 (34.46) |  | 25 (16.89) |  |
| Days 31 - 90 | | | | | | | | | | | | | |
| 1 | 277 (37.84) | 886 (319.86) | <.001 | 0 (0) | <.001 | 122 (44.04) |  | 364 (131.41) | .001 | 97 (35.02) | .002 | 33 (11.91) | <.001 |
| 2 | 174 (23.77) | 672 (386.21) |  | 12 (6.9) |  | 96 (55.17) |  | 224 (128.74) |  | 81 (46.55) |  | 45 (25.86) |  |
| 3 | 139 (18.99) | 584 (420.14) |  | 9 (6.47) |  | 20 (14.39) |  | 99 (71.22) |  | 69 (49.64) |  | 34 (24.46) |  |
| 4 | 142 (19.4) | 959 (675.35) |  | 16 (11.27) |  | 28 (19.72) |  | 155 (109.15) |  | 96 (67.61) |  | 50 (35.21) |  |

# REFERENCES

1. Mahoney F et al. Functional evaluation: the Barthel index. *Md State Med J*. 1965;14(2):61-65. doi:10.1161/01.str.30.8.1538

2. Lawton MP et al. Assessment of Older People: Self-Maintaining and Instrumental Activities of Daily Living. *Gerontologist*. Published online 1969:179-186.

3. Pfeiffer E. A Short Portable Mental Status Questionnaire for the Assessment of Organic Brain Deficit in Elderly Patients†. *J Am Geriatr Soc*. 1975;23(10):433-441. doi:10.1111/J.1532-5415.1975.TB00927.X

4. Bergstrom N et al. A clinical trial of the Braden Scale for Predicting Pressure Sore Risk. *Nurs Clin North Am*. 1987;22(2):417-428.

5. Guigoz Y et al. Malnutrition in the elderly: the Mini Nutritional Assessment (MNA). *Ther Umsch*. 1997;54(6):345—350.

6. García González J et al. Evaluación de la fiabilidad y validez de una escala de valoración social en el anciano. *Atención Primaria*. 1999;23(7):434-440.

7. Barber JH et al. A postal screening questionnaire in preventive geriatric care. *J R Coll Gen Pract*. 1980;30(210):49-51.

8. Inouye SK et al. Geriatric Syndromes: Clinical, Research and Policy Implications of a Core Geriatric Concept. *J Am Geriatr Soc*. 2007;55(5):780. doi:10.1111/J.1532-5415.2007.01156.X

9. ICD - ICD-10-CM - International Classification of Diseases, Tenth Revision, Clinical Modification. Accessed July 21, 2021. https://www.cdc.gov/nchs/icd/icd10cm.htm
